# The future of host cell protein (HCP) identification during process development and manufacturing linked to a risk‐based management for their control

**DOI:** 10.1002/bit.25628

**Published:** 2015-07-14

**Authors:** Daniel G. Bracewell, Richard Francis, C. Mark Smales

**Affiliations:** ^1^ Department of Biochemical EngineeringAdvanced Centre for Biochemical EngineeringUniversity College LondonGordon StreetLondonWC1H 0AHUK; ^2^ Francis Biopharma Ltd.SevenoaksKentUK; ^3^ Centre for Molecular ProcessingSchool of Biosciences, University of KentCanterburyKentUKCT2 7NJ

**Keywords:** host cell proteins, process‐related impurities, biopharmaceuticals, bioprocessing

## Abstract

The use of biological systems to synthesize complex therapeutic products has been a remarkable success. However, during product development, great attention must be devoted to defining acceptable levels of impurities that derive from that biological system, heading this list are host cell proteins (HCPs). Recent advances in proteomic analytics have shown how diverse this class of impurities is; as such knowledge and capability grows inevitable questions have arisen about how thorough current approaches to measuring HCPs are. The fundamental issue is how to adequately measure (and in turn monitor and control) such a large number of protein species (potentially thousands of components) to ensure safe and efficacious products. A rather elegant solution is to use an immunoassay (enzyme‐linked immunosorbent assay [ELISA]) based on polyclonal antibodies raised to the host cell (biological system) used to synthesize a particular therapeutic product. However, the measurement is entirely dependent on the antibody serum used, which dictates the sensitivity of the assay and the degree of coverage of the HCP spectrum. It provides one summed analog value for HCP amount; a positive if all HCP components can be considered equal, a negative in the more likely event one associates greater risk with certain components of the HCP proteome. In a thorough risk‐based approach, one would wish to be able to account for this. These issues have led to the investigation of orthogonal analytical methods; most prominently mass spectrometry. These techniques can potentially both identify and quantify HCPs. The ability to measure and monitor thousands of proteins proportionally increases the amount of data acquired. Significant benefits exist if the information can be used to determine critical HCPs and thereby create an improved basis for risk management. We describe a nascent approach to risk assessment of HCPs based upon such data, drawing attention to timeliness in relation to biosimilar initiatives. The development of such an approach requires databases based on cumulative knowledge of multiple risk factors that would require national and international regulators, standards authorities (e.g., NIST and NIBSC), industry and academia to all be involved in shaping what is the best approach to the adoption of the latest bioanalytical technology to this area, which is vital to delivering safe efficacious biological medicines of all types. Biotechnol. Bioeng. 2015;112: 1727–1737. © 2015 The Authors. *Biotechnology and Bioengineering* Published by Wiley Periodicals, Inc.

## Introduction

During manufacturing of therapeutic proteins destined for use in the clinic using cell‐based systems, the product itself must be purified from any cell‐based impurities to an acceptable level before administration in the clinic. Here, we focus upon impurities that may be derived from mammalian expression systems (e.g., Chinese hamster ovary [CHO] cells), whereby not only is the product of interest secreted into the cell culture fluid that is collected for harvest, but host cell proteins (HCPs), nucleic acids, lipids, and other cellular material that may be released into the culture media along with product impurities (Guiochon and Beaver, 2011). HCPs (e.g., see (Shukla et al., 2008)) and DNA (e.g., see (Zhang et al., 2014a)), in particular, must be monitored and in the final product, the general guidelines are that there should be total amounts of less than 100 ng/mL and 10 ng/dose of these impurities respectively (Chon and Zarbis‐Papastoitsis, 2011). However, this amount might be unacceptable for a particular HCP in terms of risk or product degradation, for example, residual proteases (Gao et al., 2011; Robert et al., 2009) that could potentially reduce the amount of active drug present or in the worst‐case scenario lead to the development of immunogenic forms of the product.

The philosophy at the core of downstream processing for biologics is the use of orthogonal purification steps, that is, separations based on a series of differing properties such as charge followed by hydrophobicity. This arises because of the need to separate product from the diverse spectrum of nucleic acid, lipid, and protein (the host cell proteins, HCPs) impurities derived from the host. Evaluating all the HCP components individually traditionally created an unsustainable position; hence, currently, we seek summative measures based on enzyme‐linked immunosorbent assays (ELISAs) using antibodies raised against the spectrum of HCPs found in the host cell being used. Questions have long been raised over issues associated with this approach which will be the subject of further attention in this review; at this point, these issues can be summarized as concerns as to whether all HCPs are being measured (i) at all and (ii) with sufficient sensitivity. This is an even more difficult question than it may first appear to be as all HCPs are not considered to be of equal importance (due to the risk associated with each, a concept developed in this review); therefore, the desired measurement sensitivity required for an individual HCP varies accordingly.

Our ability to directly address this measurement challenge has grown rapidly in the last decade. The demands of proteomics have driven a proliferation in bioanalytical approaches with ever increasing sensitivity, but which technologies can bring the greatest added value to this sector? The challenge is to understand where this analytical technology may best be deployed to improve the safety and efficacy of biologics during their development and manufacture. This includes consideration of high‐resolution methods suited to the research laboratory environment which may aid the creation of product and process understanding to those to be consistently used for process monitoring during manufacturing and for product release criteria. This has not gone unnoticed by the community as testified by the literature reviewed here and considerable conference activity, a good recent example of which is BEBPA's first Annual European HCP Workshop May 15–16, 2014, Dubrovnik, Croatia.

The presence of HCPs in biotherapeutic recombinant proteins destined for the clinic presents a risk to the patient if not mitigated against (Huang et al., 2009, Wang et al., 2009; Zhu‐Shimoni et al., 2014). One of the major risks that HCPs present is that of potential immunogenicity, although current approaches to monitor and measure HCP content in biologics manufactured from cell expression systems do not provide the ability to judge the risk of the presence of a particular HCP or group of HCPs. Very recently, work has emerged that has begun to address this important issue whereby in silico analysis can be undertaken in order to score the relative risk of specific HCPs (Bailey‐Kellogg et al., 2014) and others have also raised the potential for risk assessment of HCPs (Aboulaich et al., 2014; Zhu‐Shimoni et al., 2014). However, estimating relative risk alone may not be sufficient. In order to utilize such information to reduce the HCP risk, one must be able to not only identify those HCPs that are present during bioprocessing and in the final product but also the amounts of these that are present.

**Figure 1:**
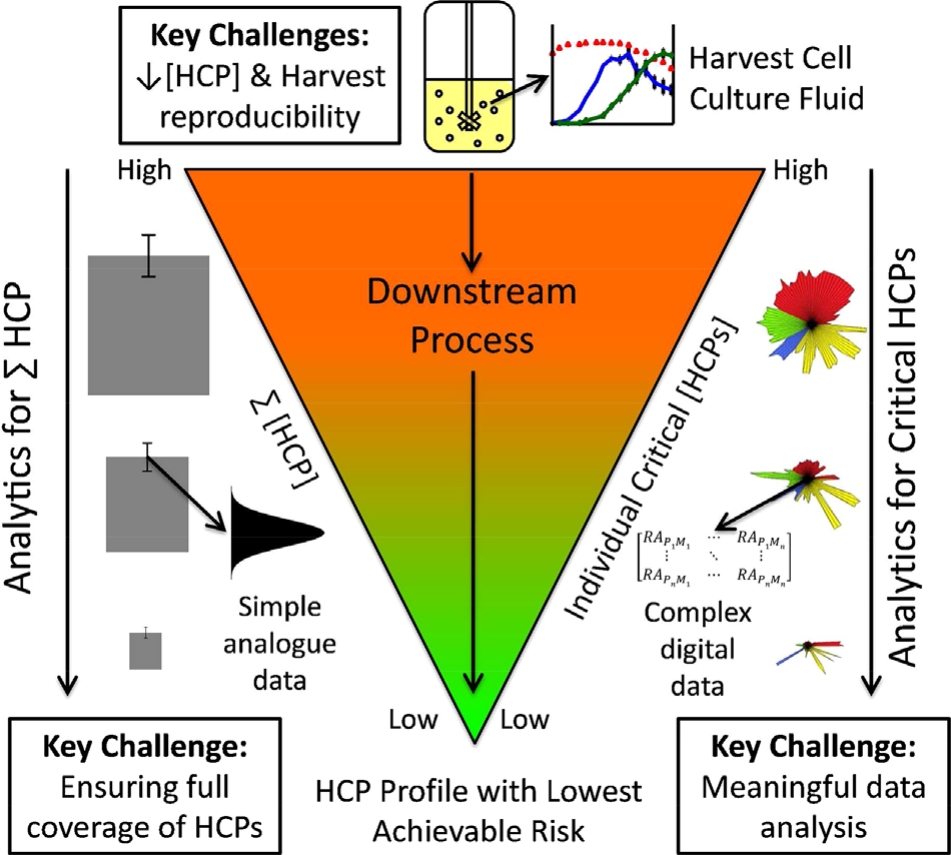
Overview of the HCP landscape in the context of biologics manufacture. The left side of the figure shows a typical ELISA‐based approach where a summed value of the HCPs detected by the polyclonal antibody used is generated with a probability of error (usually based on a Gaussian distribution as indicated). On the right side, a mass spectrometry driven approach is depicted indicating the far larger datasets of multiple species in a first mass spectra which themselves be further fragmented to create spectra for identification. The differing challenges for these differing approaches are highlighted.

**Figure 2:**
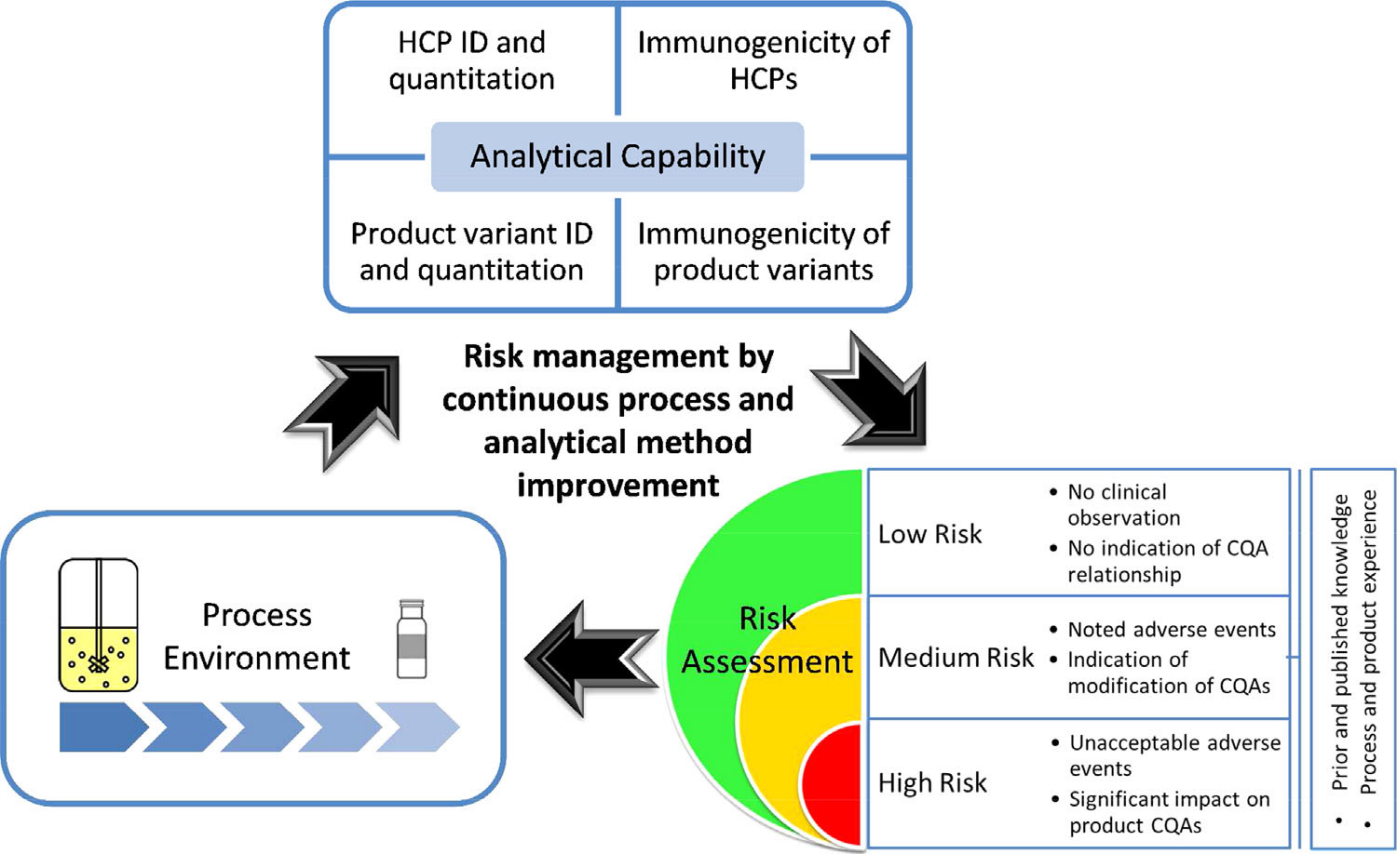
The risk assessment continuum is presented in the context of biopharmaceutical process development. In the risk assessment phase, risk is identified and assessed using prior and existing knowledge to identify which parameters may impact the critical quality attributes (CQA). Then a risk mitigation or reduction action is taken. This could be acceptance of the risk or process modification to ensure risk reduction. Then the risk assessment is repeated to determine if acceptable or not. As indicated, this is all mediated by the organization's analytical capability.

Further complicating any element of HCP risk is the nature of the product itself. A number of reports have now shown that many of the HCPs that are found in the elutes of chromatography steps, particularly protein A affinity chromatography for the purification of antibodies, are co‐purified with the product due to interaction with the product itself (Levy et al., 2014; Sisodiya et al., 2012; Tarrant et al., 2012). This “piggy backing” of HCPs through a purification process on target recombinant protein products will be product and bioprocess condition specific and, hence, the analysis of HCP risk should include consideration and knowledge of the following: (i) the target recombinant product itself; (ii) the HCPs present in the culture harvest supernatant; (iii) the bioprocess and downstream conditions to be utilized; and (iv) the risk of any given HCP or HCP group (and knowledge of a critical threshold that should not be exceeded for such HCPs/groups) remaining in the final formulated product. Although we focus the discussion here on Chinese hamster ovary (CHO)‐derived products and HCPs, such an approach could be applied to the HCP profile from any expression system currently utilized (e.g., bacterial, fungi, insect cells, plant cells). An overview of the route to the desired HCP profile, considering risk and different analytical technologies, is depicted in Figure 1. How this links to risk analysis is described further below and in Figure 2.

## Analytical Technologies for HCP Analysis

### Immunodetection‐Based Methods

The most common method for the monitoring, detection, and measurement of HCPs during bioprocessing manufacturing and in final biotherapeutic protein formulations destined for the clinic is that of ELISAs (Shukla et al., 2008; Tscheliessnig et al., 2013; Wang et al., 2009; Zhu‐Shimoni et al., 2014). These high throughput, high sensitivity, and highly selective assays have proved invaluable tools for the industry to monitor HCP amounts in process development, manufacturing and in final product formulations, and are likely to remain an important analytical tool in HCP analysis. Typically, an ELISA is established using null host cell line isolates to immunize animals and generate polyclonal antibodies (Tscheliessnig et al., 2013; Zhu‐Shimoni et al., 2014). The assumption in this approach is that the null cell line HCP profile will be similar to the recombinant protein producing cell lines derived from the same host and as such antibodies raised are likely to represent the HCP pool in the recombinant cell line fermentation harvest material (Tscheliessnig et al., 2013; Zhu‐Shimoni et al., 2014). The fact that the majority of HCPs in cell culture harvest material appear to be intracellular in nature supports this assumption. The power of the use of ELISA for the monitoring of HCPs during process development, validation, and product testing, along with the potential limitations, has been recently reviewed (Zhu‐Shimoni et al., 2014) and others have reported on efforts to increase the throughput of such immunoassay‐based methods (Heo et al., 2014). Limitations of ELISA are that in any anti‐HCP antibody pool, there are not antibodies that cover the entire spectrum of HCP species that may potentially be present and that very weakly immunogenic, or non‐immunogenic species will not be detected. Further limitations of ELISA are described by Zhu‐Shimoni et al. (2014), who also highlight the need for the use of orthogonal methods to ELISA for the measurement, monitoring, and identification of HCPs, particularly if one wishes to undertake a risk‐based assessment to identify critical HCPs. We also note that there have been reports of a high prevalence of anti‐CHO HCP antibodies in human serum in which there has been no known exposure to CHO‐derived biotherapeutic proteins, questioning the sensitivity or reliability of immunological‐based systems for detecting HCPs of high risk (Xue et al., 2010).

Any orthogonal techniques to measure and identify HCPs should ideally be able to (i) detect protein concentrations across a wide dynamic range, from very low concentrations of individual impurity proteins to those of a much higher concentration, (ii) follow the changing population and concentrations of HCPs throughout a bioprocess, (iii) monitor/measure multiple protein analytes simultaneously, and (iv) monitor/measure low concentration HCPs when “swamped” by high concentration of the target recombinant protein molecule. If a risk‐based assessment of HCPs is also to be undertaken then such methods should also be able to identify those HCPs present at any stage of the bioprocess to allow such an assessment to be undertaken. As ELISA is not able to provide information on those HCPs present in any given sample, or how the population of HCPs present changes throughout a process, additional methods to complement ELISA clearly need to be adopted if a more rigorous analysis of HCPs is to be undertaken or required by the regulatory authorities.

A number of alternative or orthogonal analytical approaches are currently used to complement ELISA measurement and monitoring of HCPs, although these are not universally applied to HCP analysis during process development or validation (e.g., Capito et al., 2013; Dickerson et al., 2011; Rey and Wendeler, 2012). The simplest are probably the use of 1D‐ and 2D‐polyacrylamide gel electrophoresis (1D/2D‐PAGE), together applied to investigate HCP dynamics (Hogwood et al., 2013; Jin et al., 2010; Tait et al., 2012; Valente et al., 2014). 2D‐PAGE is widely used for the monitoring of HCPs during process development, particularly the approach of 2D‐DIGE, whereby multiple samples can be compared on the same gel to identify those HCPs that are present or increased/decreased throughout a process (Grzeskowiak et al., 2009; Jin et al., 2010). However, PAGE methods for the detection of specific proteins are unable to detect low abundance HCPs and many HCPs can be masked by the presence of the target recombinant protein or product impurities. 2D‐PAGE coupled with mass spectrometry analysis of excised protein spots can be used to identify particular spots on gels but again this relies upon detection of the spots of interest and the sensitivity of the approach. The sensitivity of different analytical techniques that might be applied to HCP analyses is described by Tscheliessnig et al. (2013). Western blotting of 1D and 2D gels can also be used to detect and determine those HCPs present, but again as for ELISA, is limited by the pool of anti‐HCP antibodies used.

### The Emergence and Rise of Mass Spectrometry‐Based Methods

A number of other recent orthogonal approaches to ELISA have also been suggested and have recently been reviewed elsewhere (Hogwood et al., 2014; Tscheliessnig et al., 2013). However, the analytical technology that is emerging as the major orthogonal technology to ELISA is mass spectrometry (Doneanu et al., 2012; Joucla, 2013; Zhang et al., 2014b). Mass spectrometry requires a highly skilled operator and access to expensive equipment; however, the majority of analytical laboratories now contain high precision mass spectrometry technology. The power of mass spectrometry is the ability to monitor and identify multiple protein analytes in the same sample rapidly and in a high throughput manner, although obtaining absolutely quantitative data on such proteins using mass spectrometry for multiple analytes remains a challenge. Nevertheless, mass spectrometry offers the opportunity to not only monitor and measure the host cell protein and product impurity profile but the ability to identify what is, and is not, present in any particular sample, including low abundance proteins. As such, liquid chromatography coupled tandem mass spectrometry (LC‐MS/MS) has now been applied to the rapid monitoring of HCPs (Doneanu et al., 2012). Technologies used in wider proteomic studies, such as the labeling of peptides by methods such as iTRAQ, can enhance the coverage of HCPs detected beyond that using a standard 2D‐PAGE approach (Tscheliessnig et al., 2012).

Recently, others have investigated using HCP enrichment strategies combined with LC‐MS/MS to improve the detection of HCPs from antibodies generated from mammalian cell expression systems (Thompson et al., [Link]). Using such an approach, 19 HCPs were detected in a therapeutic antibody preparation compared to a single HCP identified without the enrichment strategy. This study highlights the fact that with current ELISA and MS approaches potentially important information about the HCP profile and what this contains is likely to be missed and cannot be included in any risk‐based assessment of the HCP profile remaining in a recombinant therapeutic protein preparation. Others have reported the use of mass spectrometry as an orthogonal method to ELISA in undertaking comparability studies of HCPs in drug substances before and after process change (Reisinger et al., 2014). When analyzing purified monoclonal antibody samples, Doneanu et al. (2012) identified 33 HCPs by 2D liquid chromatography coupled to mass spectrometry. Such HCPs in the purified therapeutic protein could be considered critical HCPs, although this information alone is not sufficient to label these as such and further information (e.g., potential immunogenicity, enzymatic/protease activity) needs to be considered to fully evaluate the risk of a given HCP being present. The potential of mass spectrometry for monitoring HCPs during process change has also been demonstrated by Zhang et al. (2014a) who tracked individual HCPs from the cell culture harvest fluid through to a protein A elute pool and in a number of cases even further downstream, identifying approximately 500 HCPs in the cell culture fluid and following these until no HCPs were identified in the final cation‐exchange chromatography elute pools of the purification process. These authors estimate that the individual HCP quantitation limit using the MS system they describe was approximately 13 ppm. As such, the mass spectrometry configuration used here will still not provide full detection of all HCPs present and the limit of detection is protein and/or peptide specific.

### Connecting Improved Analytical Capability to Product and Process Characterization

In identifying HCPs for the purpose of risk assessment, it is important to consider the nature of the HCP itself. Although immunogenicity is an obvious risk factor, intracellular and secreted proteases may also be present that can result in the enzymatic degradation of recombinant therapeutics (Dorai and Ganguly, 2014) during fermentation or subsequently beyond if not completely removed during downstream processing. In this case, it may be that the HCP does present an immunogenic risk; however, it is also possible that such proteases may themselves not be potentially problematic, but the fact that the presence of these could result in the generation of degraded product which could be inactive or in the worst case scenario, harmful, that presents the risk. The ability of new analytical technologies to map and monitor large numbers of HCPs, therefore, offers the potential to completely re‐think how we use such information in monitoring, process development, and in risk assessment. But how do we envisage such information be used in the industrial sense to undertake risk assessment, inform process development, and predict or reduce the potential risks to the patient?

### Process Considerations

The diversity of proteins contained within the HCP profile manifests different challenges for the process; many are enzymes and, therefore, may catalyze degradation or other undesired alterations to the product (e.g., reductases' Kao et al., 2010; Trexler‐Schmidt et al., 2010). Equally, proteins may recognize and bind to certain epitopes on the product molecule (e.g., chaperone proteins) giving rise to the possibility of HCPs that are associated with the product molecule being carried through the process. This is a phenomena now known to be the primary reason for HCPs making their way through protein A affinity chromatography (Nogal et al., 2012; Tarrant et al., 2012).

Recognizing these process associated risks for HCPs is important but the separation of perceived or theoretical risks for a given product from those actual risks where resource should be devoted to understand and mitigate such issues is central to effective process development and subsequent monitoring and control. The critical question, therefore, is how such understanding can be most rapidly acquired for any given manufacturing process to provide safe and efficacious medicines.

As identified in the analytical section of this review, the monitoring of host cell proteins remains a significant challenge in the production of biopharmaceuticals. In the absence of a single, simple, ready to deploy assay for the monitoring of the complete spectrum of these components, multiple methods are required. Even so, there can remain unrecognized risks associated with these components. Particular concern surrounds those components which may be highly active in the body such as cytokines and highly immunogenic species that present significant risk to the patient even at very low levels (Huang et al., 2009). Without high‐end analytical approaches for such HCP components, these specific issues can be hard to identify and measure with sufficient sensitivity. It has been traditionally viewed that it is not cost effective or robust to routinely use such techniques for process development and monitoring activities; therefore, the strategy used is to minimize these components to below accepted total HCP levels.

To select and prioritize assay deployment, it is critical to be aware of where components are removed during the process sequence. From a regulatory point of view, it is also an expectation that one can describe the function of each operation in terms of impurity removal. Table I provides a short summary of the issues associated with host cell proteins during manufacture that have come to light in recent years showing the complexity and challenges involved around removing HCP components where we have created a classifications structure.

**Table I:** Selected recent publications in characterization and understanding of process‐related impurities

Subject matter	Publications	Author's affiliation
Identification of critical HCPs	Levy et al. (2013), Aboulaich et al. (2014), Eon‐Duval et al. (2012), Wang et al. (2009), Valente et al. (2013), Beatson et al. (2011), Bomans et al. (2013)	Genentech, MedImmune, Uni. Delaware, Merck Serono, Pfizer, Kings, Roche, Merck (US)
HCP Process interactions	Shukla et al. (2008), Hunter et al. (2009), Aboulaich et al. (2014), Hogwood et al. (2013), Nogal et al. (2012), Tait et al. (2012), Tarrant et al. (2012), Trexler‐Schmidt et al. (2010), Joucla et al. (2013), Levy et al. (2014), Gagnon et al. (2014)	Bristol‐Myers Squibb, Pfizer, MedImmune, UCL, Uni. Kent, Genentech, Bordeaux Uni., BTI Singapore
HCP‐associated product damage	Kao et al. (2010), Dorai et al. (2011), Sandberg et al. (2006), Trexler‐Schmidt et al. (2010), Gao et al. (2011), Robert et al. (2009)	Genentech, Centocor, Biogen Idec, Biovitrum, Merck Serono

### Identification of Co‐Eluting HCPs

This issue is deserving of specific attention as until recently, it was poorly described in the literature. Publications by Shukla et al. (2008) and Shukla and Hinkley (2008) led the way, showing the importance of wash steps in disrupting HCP interactions in the purification of monoclonal antibodies via protein A affinity chromatography, while Pfizer highlighted a similar issue in an *E. coli*/hydrophobic interaction chromatography step (Hunter et al., 2009) and subsequently, the issue was followed up by several other groups (Gagnon et al., 2014; Nogal et al., 2012; Tarrant et al., 2012). Although the issue seems somewhat specific to the product and the chromatographic resin backbone used, there are some generic observations in the fact that there are clearly strong interactions between certain host cell components and the product which increase the likelihood of those impurities being co‐purified. Levy et al. (2014) have used antibody coated resins to study which HCP components are most likely to behave in this manner; they observed some degree of similarity between different antibody products and the respective host cell proteins but also significant differences. Gagnon et al. (2014) sought to identify the causative agents, identifying chromatin as a strong binder of protein A chromatography resins and giving rise to difficulties controlling the performance of this step. Such insights are directly applicable to process development and risk management.

## Regulatory Guidance and Risk Evaluation

The International Conference on Harmonization of Technical Requirements for Registration of Pharmaceuticals for Human Use (ICH) guidance Q9 Quality Risk Management outlines the key principles in understanding and managing the risk associated with the manufacture and supply of pharmaceutical products. This guidance outlines the key principles of risk assessment (identification, probability, and severity) coupled to risk management (reduction and mitigation). In terms of host cell proteins (HCPs), it is an extremely complex proposition to adequately define and control the risk factors when considering the implications of HCPs and the potential for impact on product critical quality attributes (CQAs) as well as product quality attributes (PQAs). Both are defined in ICH guidance Q8 (R2). CQAs are “physical, chemical, biological, or microbiological properties or characteristics that should be within an appropriate limit range or distribution to ensure desired product quality.” PQAs are described as “molecular or product qualities that define the quality of the product.” This basically is a methodology for evaluating the product and process‐derived components that are present in the material administered to the patient. The determination of the risk/benefit profile of a product is vital—does the benefit to the patient outweigh the risks? The difficulty is of course understanding the risk and managing the uncertainties, and the knowledge gaps. Risk is defined on the basis of occurrence or harm and severity of that harm (ICH Q9). In the case of pharmaceutical product development, a PQA must be assumed to be critical until sufficient data are generated. Managing the uncertainty is a challenging task but vital in managing the risk of adverse events to patients. The assumption of criticality, and by definition a need for control is a philosophy that prioritizes patient safety, is defined as a “precautionary principle” (Martuzzi and Tickner, 2014). This principal when applied to therapeutic products is captured in the statement “rather than presume that specific substances are safe until proven dangerous, the precautionary principle establishes a presumption in favor of protecting the patient health in the face of uncertainty” (Rosenberg et al., 2012). In order to manage and mitigate the risk factors, understanding the critical elements is a key first step. The principle mechanism of concern for HCPs is twofold; firstly, potential immunogenicity, and secondly, the concern that HCPs could act as agents of modification of the desired product.

Given that HCP is a PQA and a CQA until proven safe the first logical step in the risk assessment process is the establishment of an appropriate limit, range, or distribution to ensure consistent delivery of a product delivering desired product quality. In terms of HCPs, the assumed straight‐forward approach is to measure directly the HCP level by a proven method and set appropriate limits. The question is which one method achieves this for HCPs, given the potential for heterogeneity of the HCP population and the possibility of product/HCP binding. The next step in risk assessment is development of knowledge to allow for science‐driven assessment and to minimize potential uncertainties. The potential complexity of the HCP population is a significant factor. For example, it is estimated that the Chinese hamster genome contains 24,044 genes of interest in relation to HCP expression (Lewis et al., 2013) and *E. coli* has approximately 4,300 genes (Blattner et al., 1997). Just at a genomic level, the complexity is already potentially overwhelming. It is not feasible or possible to consider each gene expression product in isolation. This is then further complicated at the proteome level when the expressed proteins are exposed to additional modification. It has been reported that one protein was found to have more than 20 different modified forms (Godovac‐Zimmermann and Brown, 2001). Examples of the types of modifications that can generate HCPs with different biochemical properties, and thus, further complicate the HCP evaluation process, include glycosylation, phosphorylation, acetylation, sumoylation, and truncations. This is most likely an underestimation; a well characterized monoclonal antibody theoretically has the potential for (9,600)^2^ post‐translational modifications (Kozlowski and Swann, 2006). So, what chance is there of the identification of 24,044 × (9,600)^2^ potential variants? How are the critical HCPs selected and evaluated, and most importantly, controlled?

### Establishing HCP Impurity Specifications

The “traditional” practice is to set a target of 100 ng/mg therapeutic protein as an acceptable limit for residual HCP. A key question, therefore, is what forms/species of HCPs comprise this 100 ng/mg. The characterization of all residual HCPs typically is a difficult and complex analytical challenge as previously highlighted. It is unlikely that one method will be sufficient; this is especially true in the process characterization phase of the product development life cycle. Consequently, the most significant deliverable is knowledge, of both product and process. In terms of HCPs, this would equate to characterization of the types and masses of individual HCPs to the extent where potential interactions with product or patients could be assessed. It is not just a case of making a measurement of generic mass of non‐product‐derived proteins and, hence, then by definition other or “HCP” proteins present in the process samples. Rather, an understanding of the dynamics of the HCP protein population through the process flow from cell substrate propagation through to final drug product is required. This, in itself, is then part of the narrative as the key element is determination of risk to product attributes due to the presence of the HCPs. So, are the HCPs in themselves potentially immunogenic and, hence, impacting on product safety? Do any of the individual proteins forming the general mass labeled HCP have the potential to modify the therapeutic product by enzymatic activity or forming an association with the product, such as hitchhiker antigens? (Hunter et al., 2009; Luhrs et al., 2009). The fundamental question is how reasonable assessments as to the potential risk can be made when considering the complexity and the potential for multiple interactions between product/process and residual HCPs. Yet, other considerations in relation to HCPs include the potential adjuvant characteristics. Such properties could result in generation of anti‐HCP antibodies or even result in anti‐product antibody formation resulting in loss of product efficacy. Both outcomes could lead to an increase in the risk of adverse events.

The above leads us to question whether a 100 ng/mg target is really acceptable? Should the definition be more specific and based upon a more detailed understanding of the HCP population present? The truth is that the actual impact of residual HCPs cannot currently be fully understood. However, the best practice is to assume a worst case scenario and act accordingly. This dictates that the lowest reasonable level of residual HCP as assessed by several methods must be the goal. It is poor science just to assume low risk because a level of 100 ng/mg has for related products been determined as safe. A further level of characterization should be attempted to develop sufficient knowledge to allow for science‐based risk assessment. It is, therefore, logical to develop the capability to characterize and quantify HCPs early in the product life cycle. These tools are needed to facilitate process development with a focus on HCP reduction and control. To properly assess the risk factors, it is important to understand the mechanisms for generation and selection of identified HCPs during the upstream and downstream processes. It is essential to understand as far as is reasonably possible the nature of the HCP population during the product development progression. For example, it is typical that the product used for pre‐clinical studies is less pure than that used for phase III and beyond (when the manufacturing process has been further developed), and is likely to have higher HCP levels and more species of HCP. At early stages, some companies may believe it is sufficient to use commercial ELISA kits and set specifications at 100 ng/mg. This approach does give a measure of a level of HCP but in reality, each process and product would benefit from its own specific HCP assay method using null cell lines (not producing product) to develop immunogen. Any anti‐HCP antibodies must be characterized to demonstrate specificity in terms of what proportion of the total HCP population is covered. A 2D gel followed by Western blotting with the anti‐HCP antibody, though highly variable and not very quantitative, is a fairly common means of demonstrating the degree of coverage. The higher the proportion of the HCP population bound by the antibodies the greater the confidence and utility in the method for specification setting and demonstration of process control. If the investment in development of such robust methodologies for HCP detection and characterization is delayed until pre‐phase III clinical trials the opportunity of knowledge generation is lost. This represents a potentially serious omission considering a primary aim in product development is the generation of sufficient knowledge to demonstrate control. In the case of HCPs, if the control is well demonstrated then routine testing may not be required on a regular basis and so consequently there is a return on early investment in HCP analytic capability for both qualitative and quantitative assessments.

### The Basis for a Risk‐Based Approach

The goal of risk evaluation is to provide a reasonable scientific basis for establishing the levels of a product attribute that would be expected, with a high degree of confidence, to have no adverse impact on product quality and, hence, performance. It is also essential that the assessment of quality characteristics should be performed throughout the product lifecycle, even if a favorable safety/efficacy profile has been established. Continuous improvement to PQAs is encouraged so as to produce the best possible product. The difficulty in particular with HCPs is the complexity and the related uncertainty factors. It is not possible to fully predict potential immunogenicity given the range of different patient factors, so, severity will always remain high. The lack of definition of specific risk does not allow for a “wait and see” approach. A proposed risk management process is summarized in Figure 2.

The ICH guidance Q6B (1999) Specifications state that test procedures and acceptance criteria for biotechnological/biological products, guides the setting of acceptance criteria and specifications in which HCPs as process (cell substrate derived) impurities are considered. This gives a very high level limit test driven approach, although in the case of HCPs, this needs further levels of consideration, principally due to the rapid developments in protein analytical characterization methods. It is far easier today to map out the complete proteome environment than it was in 1999 when the guidelines were issued. There now exists the capabilities to resolve to very low levels with new methods in development. The hope is that this will yield a better understanding and enhance the ability of predicting the immunogenicity or product modification capabilities of individual HCPs (Xing et al., 2009).

However, this still leaves the question as to how to assess the risk of the presence of very low levels of HCP impurities. A key approach is to design quality into the process rather than just performing analysis on process derived samples. This Quality by Design (QbD) approach is in alignment with the ICH guidelines 8(R2), 10, and 11 and is in essence a systematic approach to generate process and product knowledge to define an acceptable process operational or design space. It is important that the data leading to the assignment of criticality for a PQA is derived from multiple sources; these include the direct testing and characterization data sets and also literature reviews, published scientific knowledge and any available animal/human pre‐clinical and clinical studies. However, given the potential complexity coupled to the degree of uncertainty, it is generally rational to consider HCPs as a CQA. Given this definition any process parameter relating to HCP formation and removal should then automatically be designated as a critical process parameter (CPP) and controlled appropriately.

In terms of the HCP analytical tool kit and risk assessment, the key first step is to develop a holistic view of the overall cell substrate derived HCP population. In particular, development of the understanding of how changes in the expressed HCP population relate to the producer cell line is a critical element in the risk management process. The risk analysis process would then be driven firstly by the ability to detect and identify the total HCP population. This would then facilitate a process design to maximize the efficient removal of HCP species and develop a robust process capability. From this, specific HCPs of interest (those found to be associated with elevated risk) might be selected for further consideration. Utilization of polyclonal antibody‐based ELISA and Western blot methodologies are invaluable in terms of mapping the potential diversity of HCP populations from initial cell substrate through to purified Drug Substance (Krawitz et al., 2005).

### A Structured Approach to Establishing HCP‐Associated Risk

The knowledge acquired from the deployed analytical methods can be used to rank HCPs based upon the following:

1. Severity—including immunogenicity (microbial far greater than mammalian, within which rodent far greater than human or primate), biological activity (e.g., hormones), and product interactions (e.g., proteases).
2. Detection—how easily is the class of HCP or specific HCP detected and quantified?
3. Abundance—what is the amount of an HCP or HCP class?

### HCP Severity

In such a ranking, the expression system is likely to influence the determination of HCP risk. Consequently, for *E. coli* HCP, the likelihood of an immunological reaction is higher because of the higher probability of a “foreign” epitope being recognized by a T cell. The result of a first exposure to an antigen is the generation of antibodies and induction of memory cells, and this priming leads to an immune response on re‐exposure. Other immunological reactions may manifest as an acute hypersensitivity reaction leading to anaphylaxis, cytokine release syndrome, and other challenging immune responses (Huang et al., 2009; Singh, 2009). It is also important to consider that potential adjuvant activity can arise through multiple mechanisms, including the presence of microbial impurities in therapeutic protein products. Although the likelihood of this is very low, this mechanism could in fact enhance anti‐product antibodies increasing the risk of clinical complications and also reducing the product efficacy (Lundström et al., 2014). The difficulty in assessing the risk factors relating to HCPs and potential immunogenicity derived directly from the presence of HCPs themselves, and also product‐derived species arising from HCP‐mediated modifications, is the knowledge gap. How can the potential for immunogenicity be assessed? The US Food and Drug Administration Guidance for Industry Immunogenicity Assessment for Therapeutic Protein Products details a risk‐based approach to provide investigators with the tools to develop novel protein therapeutics and evaluate the potential need for tolerance‐inducing protocols when severe consequences result from immunogenicity. The key to the approach is combining analytical methods not only for direct HCP detection and quantification, but also highlights the need for methods capable of assessing antibody formation or allergic reactions to HCPs administered with the product. The analytical methods are required at the earliest stages of the product lifecycle (pre‐clinical) to allow for the monitoring of potential impact of HCPs in animal and early clinical studies.

In terms of defining risk, the initial assumptions would be that severity of the presence of HCP is set to the highest level. This uncertainty creates elevated risk driving the need for accurate measurement of HCPs; this need can only be lessened on the basis of actual knowledge (Rosenberg, 2012).

### Detection and Abundance—Generation of the HCP Polyclonal Serum

In terms of HCP, estimation of abundance is a critical knowledge defining activity which as stated is performed by ELISA; hence, generation of the polyclonal used is the singularly most important factor in the analytical method. The type of (null cell‐derived) immunogen used is critical, for example, the point in the mock process stream at which it is derived—from an early stage of the DSP, the middle or at an end stage, or a mixture/blend from multiple parts of the process? Factors such as these must be considered when developing an antibody‐based method of HCP quantification. In certain cases, the adjuvant used to immunize the donor animal has also been demonstrated to influence the affinity of the antibodies or produce a higher population of antibodies for the specific HCP (Lundström et al., 2014). From a risk assessment perspective, the criticality of the HCP analytical methods dictate that the best practices are used.

### The Impact of a Risk‐Based Approach to HCP Analysis

The combination of these factors allows for a risk assessment value to be determined. At the start of a product lifecycle, this will be anticipated to be at its highest, with the most significant knowledge gap. As methods are refined or new methods coupled to experience gained from process and product usage, the knowledge generation should close identified gaps. The ability to better access the potential for product/process/HCP interactions will be realized which is a key foundation for scientific risk‐based assessments. It is important to focus on the product being defined in the ICH Q6 sense containing both product‐derived and process‐derived impurities to enable a level of risk acceptance to be tolerated as knowledge increases. The key part of the risk management approach as delineated in ICH Q9 is the utilization of the increased knowledge/analytical methodology to seek ways of reducing the risk and developing a mitigation strategy. For HCP, a potential risk mitigation strategy could include the following:

1. Engineering of the cell substrate, purposely deleting specific genes that are not required for cell function in terms of producing the defined product, or specific deletion of genes for problematic HCPs (Humphreys et al., 2004) such as proteases that are proven to cause unwanted product modifications.
2. Improving process performance to eliminate and remove HCP impurities. This may result in lower product yields, but in terms of process priorities safety is the prime driver in process development.
3. Continuously evaluating and developing analytical methods for assessment of process performance and the impact of product purity during clinical performance, and ensuring that these data are used to refine process performance. Examples could include specifically targeting a specified HCP in order to eliminate or control to a tighter tolerance.

The emphasis is that the risk reduction strategy supports and facilitates the adequate control of the risk identified due to the HCPs. The information gathered is then incorporated into the process control strategy as this is developed in conjunction with the product manufacturing process and definition of the process design space.

However, risk can never be eliminated and instead it must be minimised by defining what is acceptable using a scientific rationale to justify the identified risk. The essential mitigation will be demonstration of control typically by specifications which define safe levels of critical HCPs (typically based upon knowledge of what has been present in clinical batches). This should be linked to process performance and ensuring the process operates within the same operation space (or a more stringent version with tighter ranges) used for the manufacturer of the clinical batches.

### The Demands of HCP Analysis for Standards and Biosimilars

The HCP population and its potential to influence product attributes is a significant consideration in terms of development of biosimilar products. Proving “similarity” is an onerous analytical challenge with such a high degree of complexity arising in product‐related impurities and HCPs. It requires the definition of the originator product alongside a proposed biosimilar to make this case. Analogous considerations must be made for the originator product where a process improvement is proposed. The creation of a reduced impurity profile product might be an option in both scenarios but will have consequences in terms of the regulators perception of such products and the amount of clinical data expected to support approval.

These are difficult propositions to answer in a generic manner and will require a case by case consideration. However, it seems a logical consideration that the fewer impurities such as HCPs the greater the positive impact of product attributes such as safety and reduction in potential immunogenicity and in these situations standards in terms of materials and analytical methods could be of great assistance. This must be balanced against the fact not all HCPs are equal in the risk they present, as we have identified. This is consistent with the intentions outlined in the ICH guidance Q5E which states “Improvement of product quality is always desirable and encouraged.”

## A Proposed Route Forward

**Figure 3:**
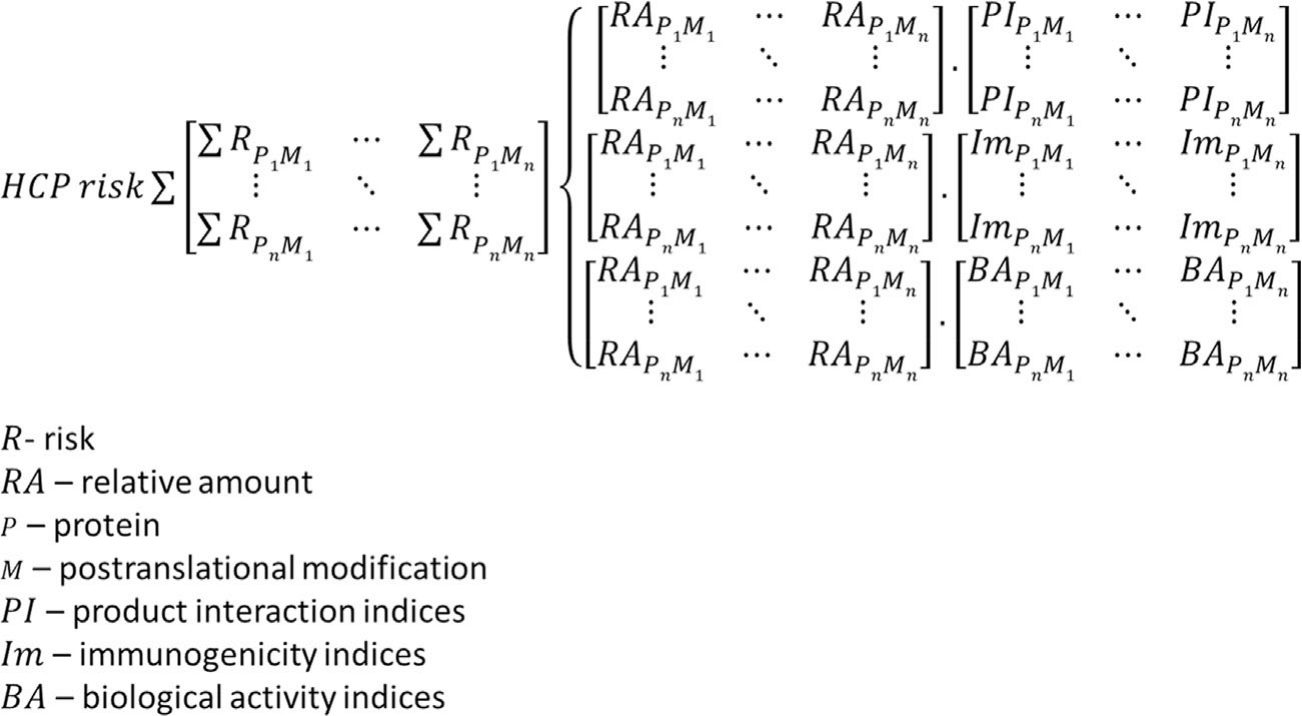
Proposed approach to a summed risk index for a measured HCP profile. In this scenario, RA would be derived from mass spectrometry‐based techniques, and the indices PI, Im, and BA would be based on agreed standards.

Combining risk indices associated with the host cell proteome suggested in Figure 3 to include immunogenicity, biological activity, and a measure of the interaction with the product during manufacture and storage, while a huge task due to the number of protein species, is computationally possible assuming the methods to calculate these indices can be determined and agreed upon. As the utility of proteomic databases grow alongside existing genomic information and algorithms to predict immunogenicity (Bailey‐Kellogg et al., 2014) and protein interactions (Wass et al., 2010) it may not be that distant an aspiration to standardise some of the matrices indicated in Figure 3 and use the input from mass spectrometry to provide quantitative indices of risk that a particular HCP profile might pose. It would be critical that such indices could be agreed upon to avoid their proliferation which would rapidly mean such an approach be rendered meaningless. This might follow an ICH model to create agreed approaches/indices, although this would require a custodian of this information which would most likely need to involve national standards laboratories. It would enable generic databases to be generated (e.g., relating to the CHO proteome) to quantify a summed HCP risk value based on mass spectrometry data from a user, hence, making it possible for information which might be regarded as commercially sensitive to remain confidential.

## Conclusion

Without knowledge and supportive data, the HCP population has to be assumed to be a critical product attribute in the assessment of risk. The only means of changing the uncertainty rating in terms of effects on product safety is a risk reduction strategy based on utilizing the best possible analytical methods as soon as is feasible in the product lifecycle development. Given the assumed success of a product then the data generated in terms of HCP type, distribution and relative abundance will prove invaluable later when assessing risk of process changes/improvements, comparability studies, and many other situations that will occur during a product lifecycle. Additionally, such analytical tools for HCP characterization and quantification are invaluable in terms of creating a defined design or control space for process operation to ensure the best possible product generation at all phases.

This work was supported by the BBSRC, EPSRC, and industrial club members under the Bioprocessing Research Industry Club (BRIC) initiative (Ref. BB/G010307/1 at Kent; BB/G010307/1 at UCL).
